# Comparison of the effects of one-level and bi-level pre-incisional erector spinae plane block on postoperative acute pain in video-assisted thoracoscopic surgery; a prospective, randomized, double-blind trial

**DOI:** 10.1186/s12871-023-02232-8

**Published:** 2023-08-11

**Authors:** Emine Nilgün Zengin, Musa Zengin, Hülya Yiğit, Hilal Sazak, Sumru Şekerci, Ali Alagöz

**Affiliations:** 1 Ankara Bilkent City Hospital, Anesthesiology and Reanimation Clinic, Ministry of Health, Ankara, Turkey; 2 Ankara Etlik City Hospital, Anesthesiology and Reanimation Clinic, Ministry of Health, Ankara, Turkey; 3 University of Health Sciences, Ankara Atatürk Sanatorium Training and Research Hospital, Ankara, Turkey

**Keywords:** Acute pain, Bi-level erector spinae plane block, Postoperative pain, Video-assisted thoracic surgery

## Abstract

**Background:**

This prospective, randomized, double-blind trial aimed to compare the postoperative analgesic efficacy of One-Level pre-incisional erector spinae plane block (ESPB) and Bi-Level pre-incisional ESPB in patients undergoing video-assisted thoracic surgery (VATS).

**Methods:**

This pilot trial was conducted between April 2022 and February 2023 with sixty patients. The patients were randomly divided into two groups. In One-Level ESPB Group (n = 30) block was performed at the thoracal(T)5 level with the 30 ml 0.25% bupivacaine. In the Bi-Level ESPB Group (n = 30) block was performed at T4 and T6 levels by using 15 ml of 0.25% bupivacaine for each level. In the postoperative period, 50 mg dexketoprofen every 12 h and 1 g paracetamol every 8 h were given intravenously (IV). Patient-controlled analgesia (PCA) prepared with morphine was applied to the patients. 0.5 mg/kg of tramadol was administered via IV for rescue analgesia. Visual analog scale (VAS) scores were recorded in the postoperative 1^st^, 2^nd^, 4^th^, 12^th^, 24^th^, and 48^th^ -hours. The need for additional analgesics and side effects were recorded. In two groups, patients’ demographics and postoperative hemodynamic data were recorded.

**Results:**

VAS scores at resting were statistically significantly higher at the 1^st^ (p: 0.002) and 4^th^ -hour (p: 0.001) in the One-Level ESPB. When the groups were evaluated in terms of VAS coughing scores, the 4^th^ -hour (p: 0.001) VAS coughing scores results were found to be statistically significantly higher in the One-Level ESPB group. In terms of VAS values evaluated during follow-up, the rates of VAS coughing score > 3 values were found to be statistically significantly lower in the Bi-Level ESPB group (p: 0.011). There was no statistically significant difference between the groups in terms of side effects, morphine consumption, and additional analgesic use (p > 0.05).

**Conclusions:**

Adequate analgesia was achieved in the early postoperative period in the group treated with Bi-Level ESPB with similar morphine consumption and side effects. This may be an advantage, especially in the early postoperative period when the pain is quite intense.

## Background

Although anatomical lung resections are traditionally performed with thoracotomy, video-assisted thoracic surgery (VATS) has become more prevalent in recent years due to the development of minimally invasive surgical procedures [1]. Compared to thoracotomy, VATS offers advantages such as reduced perioperative pain, faster recovery, and shorter hospital stay [2, 3]. While surgical trauma is minimized with VATS, certain factors like intercostal nerve injuries, muscle injuries, rib contractions, and pleural damage can still cause significant pain after thoracoscopic surgery [4]. If this acute pain is not effectively managed, it can affect the outcomes and even lead to the development of chronic pain [5–7].

In addition to systemic analgesia, various regional analgesia techniques are employed in the treatment of acute pain following thoracic surgery. Thoracic paravertebral block (TPVB) is also a commonly used technique in thoracic surgery. TPVB, which is also frequently used in VATS applications, should be applied carefully due to its proximity to the pleura [1–3]. In addition, it should be followed closely in terms of side effects due to ipsilateral sympathetic block [4].

In recent years, there has been a rapid development in plane blocks with the frequent use of ultrasound (US) in the operating room [8]. Erector spinae plane block (ESPB), described by Forero et al. in 2016, was one of them [9]. ESPB can be safely performed as it is situated at a adistance from the pleura and neuraxial structures. In addition, it is easier to apply because it is more superficial than the thoracic paravertebral area [2, 10]. In ESPB, a local anesthetic (LA) solution is injected into a virtual plane between the transverse processes and the erector spinae muscle and spreads over a variable number of vertebral levels. Large volumes of LA may be required for a successful ESPB. Considering that there is a rich vascular bed in the erector spinae muscle group, care should be taken in terms of LA systemic toxicity (LAST) [11]. There is no clear consensus in the studies conducted to determine the optimal level that can be achieved with volume expansion. The volume required to cover a dermatome has been claimed to range from 2.5 mL to 6.6 mL, with a median value of 3.4 mL [12]. In line with these studies, studies were conducted comparing the volume of LA for ESPB [13, 14]. However, in these studies, LA was administered from a single injection site. Although there are publications in which ESPB was applied at different levels in the same patient, we could not find any study comparing the levels within itself for thoracic surgery [15–17].

The hypothesis of this study is that Bi-Level ESPB, involving the administration of LA at two different levels, would result in a greater spread and thus provide more effective postoperative analgesia. Based on this hypothesis, the aim of this study was to compare the postoperative analgesic efficacy of pre-incisional One-Level ESPB and Bi-Level ESPB in patients undergoing VATS.

## **Materials and methods**

### Study design and patients

The prospective, randomized, and double-blind trial was conducted in two centers (Ankara Bilkent City Hospital and Ankara Atatürk Sanatorium Training and Research Hospital) after obtaining approval from the Ankara City Hospital Ethical Committee (IRB: E.Kurul-E1-22-2534 / 04.2022). The trial was registered on www.clinicaltrials.gov (https://clinicaltrials.gov/) under the identifier NCT05427955 on 22/06/2022 (principal investigator: Emine Nilgün Zengin, MD). All procedures performed in studies involving human participants were in accordance with the ethical standards of the institutional and/or national research committee and with the 1964 Helsinki declaration (as revised in 2013) and its later amendments or comparable ethical standards. Informed consent was obtained from all participants and was written in this study (first enrolment: 19.07.2022, final enrolment: 20.12.2022).

Inclusion criteria included age between 18 and 80 years, body mass index (BMI) between 18 and 40 kg/m^2^, American Society of Anesthesiologists (ASA) 1–3, and undergoing elective VATS. Exclusion criteria from the study were as follows: History of a bleeding disorder, chronic pain treatment, LA allergy, infection in the area where ESPB will be applied, and emergency surgery.

Patients scheduled for lung resection with VATS procedure in two centers were identified as possible candidates for the study. Patients who met the inclusion criteria stated on the day of surgery were randomized in the operating room and an appropriate block procedure was planned. All information about the study was explained in detail to all patients. The patients included in the study were given training on pain assessment and the use of the patient-controlled analgesia (PCA) device.

### Randomization and grouping

Block randomization was applied to the study. Random blocks were created for patients. Treatments were designated as O (One-Level) and B (Bi-Level). One block consisted of six randomly ordered treatment assignments (OOBOBB, BOBOOB, etc.). As patients entered the trial, they received the next treatment in the current block. This study consisted of blocks of six patients, and the number of patients assigned to two treatments in a block was never more than three. Thus, patients were randomly assigned to two groups of 30 individuals each, with an allocation ratio of 1:1. Group I included those to be administered One-Level ESPB and Group II included those to be administered Bi-Level ESPB.

#### Outcomes

In this study, visual analog scale (VAS) scores at rest(VASr) and cough(VASc) were determined as the primary outcome. Postoperative morphine consumption, additional analgesics used, and side effects were determined as secondary outcomes of the study.

#### General anesthesia

The patients’ were monitorisation was performed according to ASA standards. 0.03 mg/kg intravenous (iv) midazolam was given to the patients. After preoxygenation, 2 mg/kg propofol, 1.5 mcg/kg fentanyl, and 0.1 mg/kg vecuronium were administered for anesthesia induction. Intubation was performed with an appropriate-sized left double-lumen tube and the location of the tube was confirmed by fiberoptic bronchoscopy. Sevoflurane inhalation and remifentanil infusion (0.01–0.20 mcg/kg/min) were used for anesthesia maintenance. Sevoflurane inhalation and remifentanil infusion were adjusted to maintain the target heart rate and blood pressure within 20% from basal measurements. Biportal VATS was applied to the patients and a single chest tube was inserted.

#### Block procedures

The patients were placed in the lateral decubitus position after general anesthesia. Strict sterile conditions were provided in the area where the block was to be applied. Block applications were performed under US guidance. Linear probe and US-compatible 22 gauge and 8 mm nerve block needle were used for block application.

##### **One-level ESPB Group (n = 30)**

When the patients in the lateral decubitus position, ESPB was performed at the level of the thoracal(T)5 vertebrae. The probe was placed longitudinally, 2–3 cm lateral from the midline. Anatomical structures were seen. The needle was advanced in the caudo-cranial direction under the erector spinae muscle over the T5 transverse process with the in-plane technique. As a control, hydro-dissection was performed with 2 mL of saline. Then, 30 mL of 0.25% bupivacaine was injected into this area.

##### **Bi-Level ESPB Group (n = 30)**

In this group, the US probe was first placed over the T4 transverse process and then over the T6 transverse process, respectively. For both levels, the needle was advanced as in the One-level group. 15 mL of 0.25% bupivacaine was injected into both injection sites, respectively.

#### Analgesia protocol

Multimodal analgesia was provided by administering 100 mg tramadol and 50 mg dexketoprofen intravenously (IV) at the end of the surgery. 10 mg metoclopramide was given to prevent nausea/vomiting. In the postoperative period, 50 mg dexketoprofen every 12 h and 1 g paracetamol every 8 h were given. In addition, IV PCA prepared with morphine was administered to the patients. The PCA pump’s dose delivery was limited to administering a bolus dose of 1 mg of morphine and delivering a maximum dose of 12 mg of morphine in total within 4 h with lockout intervals of 15 min. The pain was defined with a 0–10 point (0: No pain and 10: Unbearable pain) VAS. When the VASr score was ≥ 4, 0.5 mg/kg of tramadol was administered via IV for rescue analgesia. The patients, who were followed up in the surgical intensive care unit for 24 h, were then transferred to the ward.

Block applications were performed by two anesthetists who are experienced in the use of the US in both centers. VAS follow-ups of the patients were performed by pain management nurses who did not know (blinded) the type of block applied to the patient and were not included in the study.

VASr and VASc scores were recorded in the postoperative 1^st^ hour, 2^nd^ hour, 4^th^ hour, 12^th^ hour, 24^th^ hour, and 48^th^ hour. Patients’ hemodynamic data (mean arterial pressure, heart rate, and SpO_2_), the need for additional analgesics, and side effects including allergic reactions, respiratory depression, hypotension, urinary retention, nausea-vomiting, and itching were recorded during the postoperative 24 h. In two groups, patients’ age, BMI, gender, diagnosis, and type of surgery were recorded.

#### Statistical analysis and sample size

SPSS for Windows, version 22.0 (SPSS Inc., Chicago, IL, United States) was used for data analyses. Whether the distribution of continuous variables was normal or not was determined by the Kolmogorov-Smirnov test. The Levene test was used for the evaluation of the homogeneity of variances. Unless specified otherwise, continuous data were described as median (Q1: first quartile – Q3: third quartile) for skewed distributions. Categorical data were described as a number of cases (%). Statistical analysis differences in not normally distributed variables between two independent groups were compared by Mann Whitney U. Categorical variables were compared using Pearson’s Chi-Square test or Fisher’s Exact test. A p-value of < 0.05 was considered statistically significant. Bonferroni correction was used for analysis of VAS scores, statistical significance was adjusted to p < 0.0083, due to measurements from 6 time points.

The sample size was calculated using G*Power© software version 3.1.9.2 (Institute of Experimental Psychology, Heinrich Heine University, Dusseldorf, Germany). The sample size was calculated for the Mann-Whitney U-test, which was used for testing the main hypothesis of (VASr scores in the 1st postoperative hour) of the preliminary study. Depending on the preliminary study research results (VASr scores in the 1^st^ postoperative hour) with two-sided (two tails) type I error 0.05 and power of 80% (1-β = 0.8), effect size (d) factor 0.77, should involve ≥ 58 subjects.

## Results

**Fig. 1:**
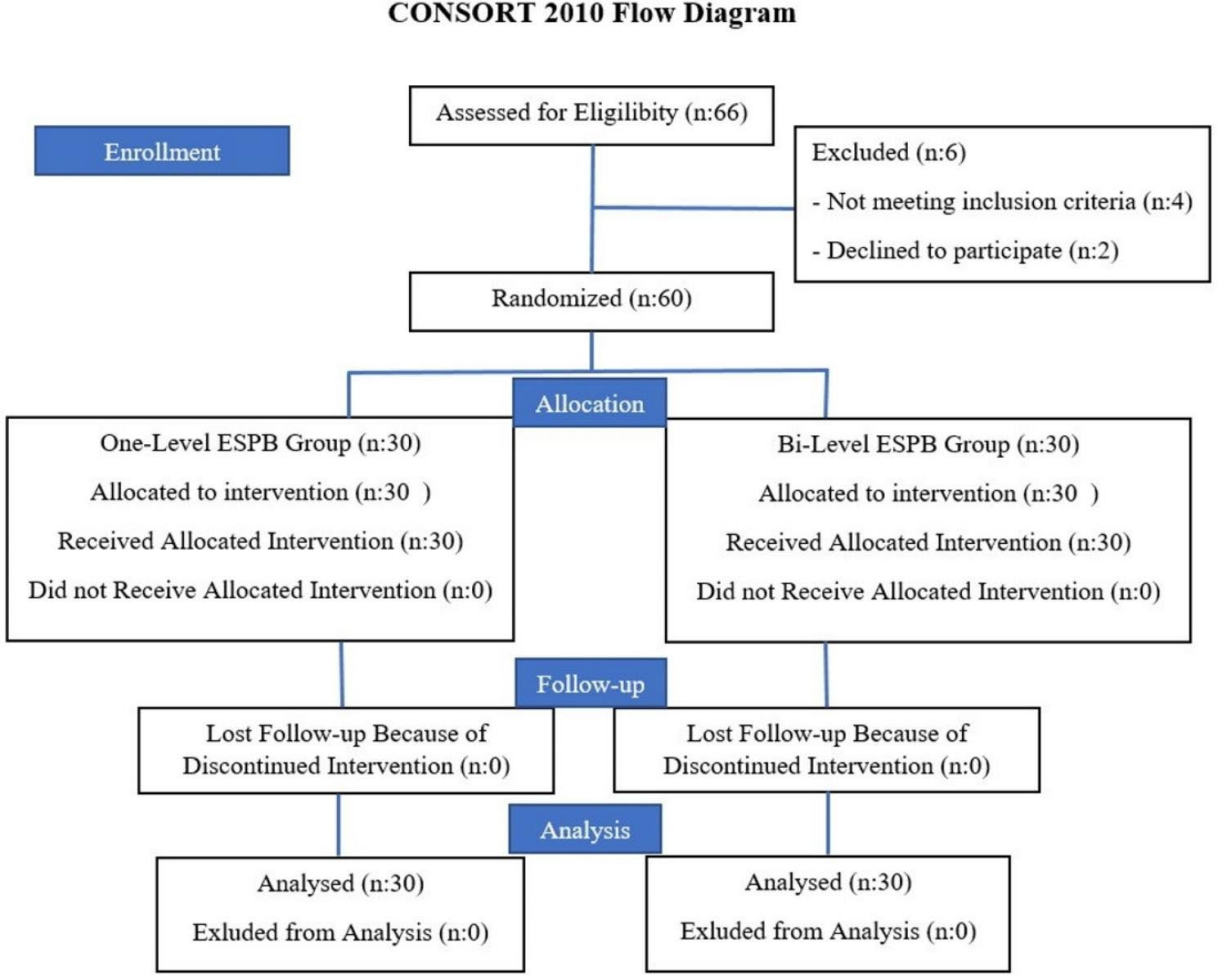
Flowchart of the patients. ESPB: Erector Spinae Plane Block

This study was conducted between April 2022 and February 2023. Sixty patients who underwent VATS were randomly divided into two groups at a ratio of 1:1. The CONSORT flow diagram was used for our study (Fig. 1).

Demographic characteristics and intraoperative findings were similar between groups (p > 0.05) (Table 1).

**Table 1:** Demographic characteristics and intraoperative findings of the patients

		One-Level ESPB (n:30)	Bi-Level ESPB (n:30)	p
Age, year ^*β*^		56.0 (35.0–64.0)	50.5 (41.0–61.0)	0.734
Gender ^*δ*^	Female	9 (30.0%)	8 (26.7%)	0.774
	Male	21 (70.0%)	22 (73.3%)	
BMI ^*β*^		25.7 (23.0-27.6)	25.8 (22.0-29.4)	0.579
Diagnosis ^*δ*^	Lung Mass	25 (83.3%)	25 (83.3%)	0.999
	Pneumothorax	5 (16.7%)	5 (16.7%)	
Surgery ^*δ*^	Wedge Resection	20 (66.7%)	23 (76.7%)	0.657
	Segmenthectomy	2 (6.66%)	1 (3.33%)	
	Lobectomy	8 (26.7%)	6 (20.0%)	
Duration of surgery ^*β*^ (min)		180 (150–210)	180 (120–210)	0.769
ASA ^*δ*^	ASA I	3 (10.0%)	6 (20.0%)	0.608
	ASA I I	11 (36.7%)	10 (33.3%)	
	ASA I I I	16 (53.3%)	14 (46.7%)	

*Continuous variables are expressed as either*^*β*^*the median (Q*_*1*_: *first quartile – Q*_*3*_: *third quartile), and categorical variables are expressed as either*^*δ*^*frequency or percentage. Continuous variables were compared with the Mann Whitney U test, and categorical variables were compared using Pearson’s Chi-Square test or Fisher exact test. Statistically significant p-values are in bold. BMI: Body mass index. ASA: American Society of Anesthesiologist; ESPB: Erector Spinae Plane Block*

There was no statistically significant difference between the groups in terms of mean arterial pressure, heart rate, and SpO_2_ (p > 0.05).

**Fig. 2:**
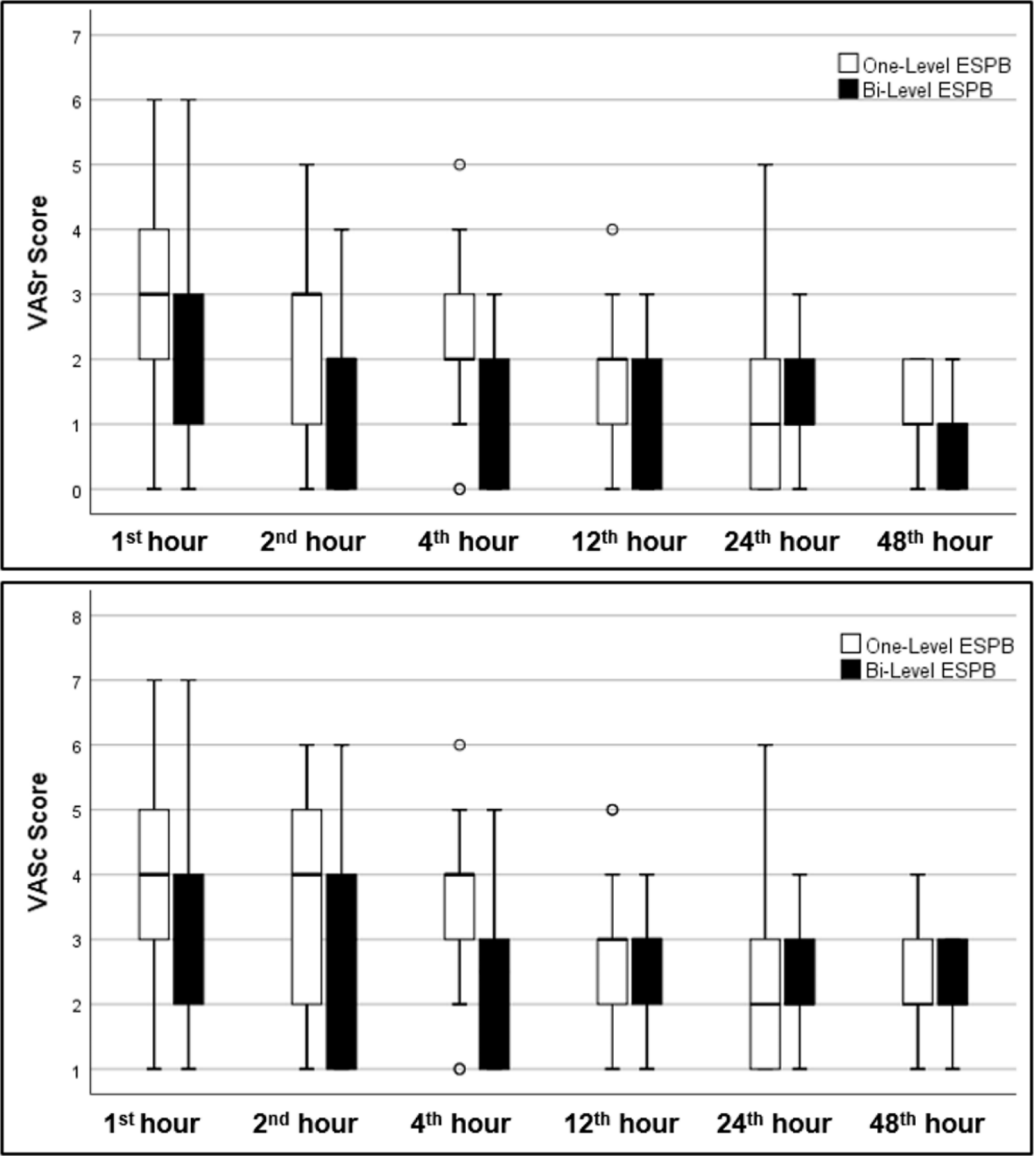
VAS scores at rest and coughing. Data are expressed as median (horizontal bars), interquartile range (boxes), and maximum and minimum values (whiskers) for the VAS scores in the 1^st^, 2^nd^, 4^th^, 12^th^, 24^th^, and 48^th^ hours. VAS: Visual analog scale; ESPB: Erector Spinae Plane Block

When the groups were evaluated in terms of VASr scores; the 1^st^ **(p: 0.002)** and 4^th^ -hour **(p: 0.001)** VASr scores results were found to be statistically significantly higher in the One-Level ESPB group than in the Bi-Level ESPB group. There was no statistically significant difference between the groups in terms of the 2^nd^ (p: 0.009), 12^th^ (p: 0.121), 24^th^ (p: 0.786), and 48^th^ -hour (p: 0.453) VASr scores (Table 2; Fig. 2).

**Table 2:** Resting and coughing VAS scores during the postoperative 48 h

	One-Level ESPB (n:30)	Bi-Level ESPB (n:30)	P*
**VASr**			
1^st^ hour	3.00 (2.00–4.00)	2.00 (1.00–3.00)	**0.002**
2^nd^ hour	3.00 (1.00–3.00)	2.00 (0–2.00)	0.009
4^th^ hour	2.00 (2.00–3.00)	1.00 (0–2.00)	**0.001**
12^th^ hour	2.00 (1.00–2.00)	1.00 (0–2.00)	0.121
24^th^ hour	1.00 (0–2.00)	1.00 (1.00–2.00)	0.786
48^th^ hour	1.00 (1.00–2.00)	1.00 (0–1.00)	0.453
**VASc**			
1^st^ hour	4.00 (3.00–5.00)	3.00 (2.00–4.00)	0.048
2^nd^ hour	4.00 (2.00–5.00)	3.00 (1.00–4.00)	0.010
4^th^ hour	4.00 (3.00–4.00)	2.50 (1.00–3.00)	**0.001**
12^th^ hour	3.00 (2.00–3.00)	3.00 (2.00–3.00)	0.455
24^th^ hour	2.00 (1.00–3.00)	2.00 (2.00–3.00)	0.835
48^th^ hour	2.00 (2.00–3.00)	2.00 (2.00–3.00)	0.523

*Continuous variables are expressed as the median (Q*_*1*_: *first quartile – Q*_*3*_: third quartile). Continuous variables were compared with the Mann-Whitney U Test*. Statistically significant p-values are in bold. P < 0.0083 according to Bonferroni correction. VASr: Visual analog scale at rest; VASc: Visual analog scale at coughing; ESPB: Erector Spinae Plane Block.

When the groups were evaluated in terms of VASc scores; the 4^th^ -hour **(p: 0.001)** VASc scores results were found to be statistically significantly higher in the One-Level ESPB group than in the Bi-Level ESPB group. There was no statistically significant difference between the groups in terms of the 1^st^ (p: 0.048), 2^nd^ (p: 0.010), 12^th^ (p: 0.455), 24^th^ (p: 0.835), and 48^th^ -hour (p: 0.523) VASc scores (Table 2; Fig. 2).

**Fig. 3:**
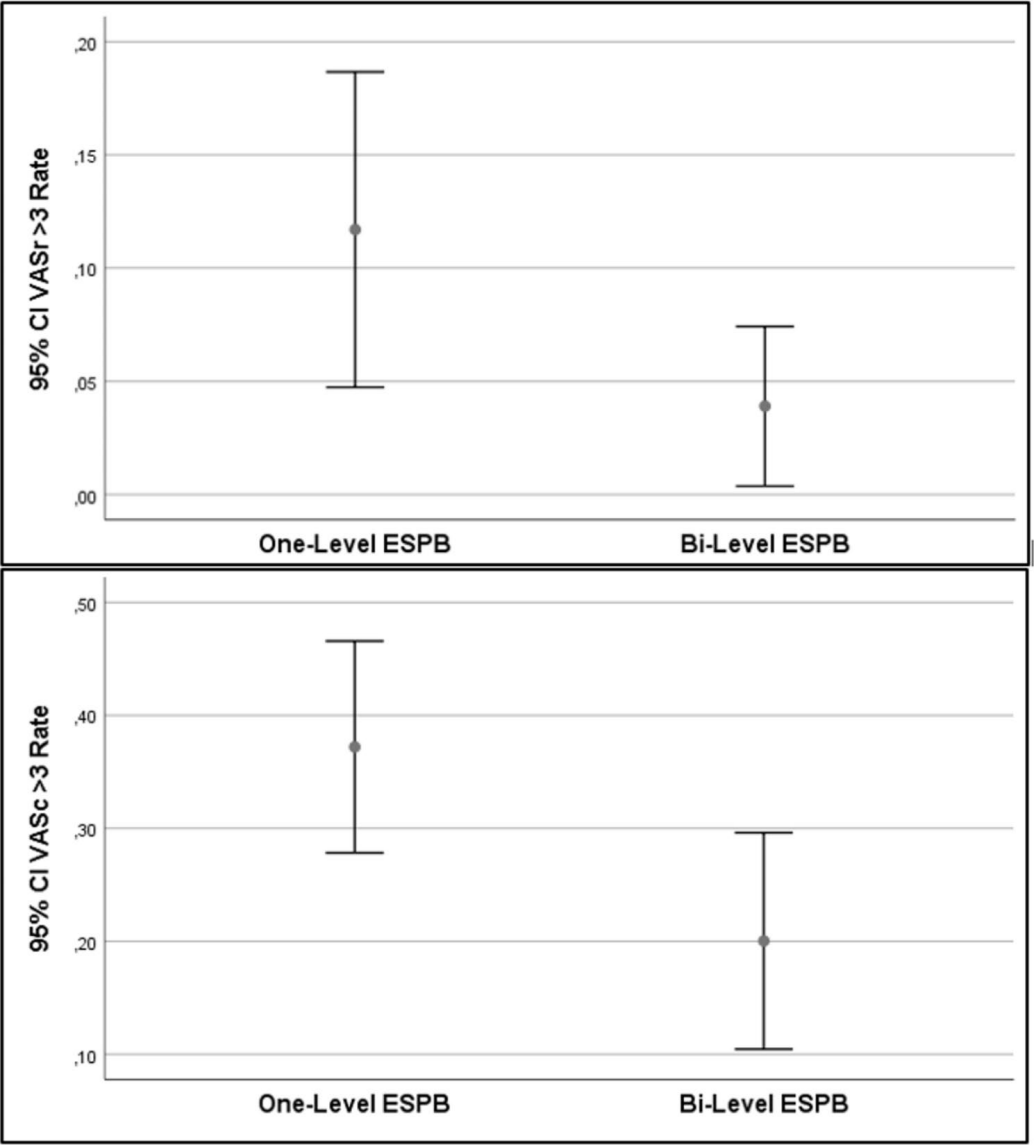
The average of those with a VAS score above 3 at all times, including the %95 confidence interval. CI: Confidence interval; ESPB: Erector Spinae Plane Block

In terms of VAS values evaluated during follow-up, the rates of VASc > 3 values were found to be statistically significantly lower in the Bi-Level ESPB group **(p: 0.011)**. On the other hand, the rates of VASr > 3 values were similar in both groups (p: 0.065) (Fig. 3) (Table 3).

**Table 3:** Rates of VAS scores > 3 values evaluated at 6 time periods

	One-Level ESPB (n:30)	Bi-Level ESPB (n:30)	p*
**VASr (> 3/10)**	0.00 (0.00-0.17)	0.00 (0.00-0.00)	0.065
**VASc (> 3/10)**	0.42 ( 0.13-0.50)	0.20 (0.00-0.33)	**0.011**

*Continuous variables are expressed as the median (Q*_*1*_: *first quartile – Q*_*3*_: third quartile). Continuous variables were compared with the Mann-Whitney U Test*. *Statistically significant p-values are in bold. VASr: Visual analog scale at rest; VASc: Visual analog scale at coughing; ESPB: Erector Spinae Plane Block*

When the patients were evaluated in terms of side effects (allergic reactions, respiratory depression, hypotension, urinary retention, nausea-vomiting, and itching), only nausea was observed. There was no statistically significant difference between the groups in terms of side effects, morphine consumption via PCA, morphine milligram equivalent consumption, and additional analgesic use (p > 0.05) (Table 4).

**Table 4:** Morphine consumption, MME consumption, additional analgesic use, and side effects during the postoperative 24 h

	One-Level ESPB (n:30)	Bi-Level ESPB (n:30)	p
Morphine Consumption via PCA *(mg)*^*β*^	12.0 (8.0–23.0)	10.0 (6.00–13.0)	0.113
MME consumption *(mg)*^*β*^	13.5 (8.00-25.7)	10.5 (6.00–15.0)	0,055
Additional Analgesic Use *n (%)*^*δ*^	11 (36.7%)	5 (16.7%)	0.080
Nausea *n (%)*^*δ*^	2 (6.66%)	1 (3.33%)	0.999
Vomiting *n (%)*	-	-	-
Allergic reactions *n (%)*	-	-	-
Respiratory depression *n (%)*	-	-	-
Hypotension *n (%)*	-	-	-
Urinary retention *n (%)*	-	-	-
Itching *n (%)*	-	-	-

*Continuous variables are expressed as either the median (Q*_*1*_: *first quartile – Q*_*3*_: *third quartile), and categorical variables are expressed as either*^*δ*^*frequency or percentage. Continuous variables were compared with the Mann Whitney U test, and categorical variables were compared using Pearson’s Chi-Square Test or Fisher Exact Test. ESPB: Erector Spinae Plane Block; MME*: *Morphine milligram equivalent; PCA: Patient-controlled analgesia.*

## Discussion

The results of ESPB application with One-Level and Bi-Level injection techniques in patients who underwent VATS due to anatomical lung resection showed that analgesia levels were more acceptable in patients who underwent Bi-Level ESPB, particularly in the early postoperative period. Bi-Level ESPB group had lower median pain scores at the 1^st^, and 4^th^ hours compared to the One-Level group. Additionally, comparable results were observed in One-Level and Bi-Level block applications in terms of cumulative morphine consumption and side effects.

In recent years, thoracic wall blocks, which are increasingly used in minimally invasive surgeries such as VATS, have become an important focus of attention [4]. This also explains the increasing use of less invasive blocks, such as fascial blocks, in clinical practice, as they are also suitable for enhanced recovery after surgery (ERAS) protocols [18]. Although there are still issues that need to be explained about the mechanism of action in many areas, providing effective analgesia with fewer complications and ease of application than central blocks makes these blocks interesting. ESPB, which has been applied for the first time in patients with thoracic neuropathic pain, is widely used among these plane blocks and gives notable results [9, 19, 20].

One of the most important question marks about the ESPB block is the determination of the optimal volume to be applied and its spread. It is thought that the analgesic effect will also be related to the amount of volume to be used and more effective analgesia can be achieved as the volume increases [21]. With the ESPB application, the LA solution is spread craniocaudal. But there are conflicting results as to reach how it can the paravertebral space. It has been argued that the most likely pathways for its entry into the paravertebral space include openings in the retinacular fascia, dorsal branches in the posterior thoracolumbar fascia, intertransverse connective tissue complex, and openings at the transition points of the accompanying vascular structures [22–26]. It is assumed that these channels do not allow fast volume pass. This allows for a gradual leakage of LA [27].

Ivanusic et al. [28] applied 20 mL of methylene blue at the T5 level in their cadaveric study and evaluated its spread. They stated that there was not sufficient spread to the paravertebral area and the anterior region of the transverse process. Adhikary et al. [23] stated that in their radiological and cadaveric study with single-injection retrolaminar block and ESPB applications, LA provides epidural and neural foraminal expansion along several levels centered around the injection level, and therefore it can be expected to have clinical effects similar to thoracic paravertebral block. Elsharkawy et al. [29] stated that many factors are effective in LA distribution in ESPB block. Tulgar et al. [30] reported that the LA injection point, LA volume, and length of the transverse process affect the distribution of LA and block efficacy. Tulgar et al. [31] reported in another study that sometimes larger dermatomal sensory blocks can be created with small volumes ESPB block compared to large volumes, and different sensory blocks can be reported even when the same volume is injected at the same level. When the individual anatomical differences in the spread of LA are added to this situation, it can be thought that a single-point injection may have a more limited effect than Bi-Level or multiple injections. There are limited studies on this subject in the different situations [15–17, 26]. Tulgar et al. [26] observed that Bi-Level ESPB provided effective analgesia in a study of thoracotomy cases they reported. Zheng et al. found that Bi-Level ESP block provided a higher rate of coverage of the surgical incision by the sensory block when compared with the One-Level method, without increasing the incidence of procedure-related complications [32].

Since studies showing that Bi-Level block is more effective are limited, the mechanism by which effective analgesia occurs has not been clarified. Possible mechanisms can be explained by anatomical, cadaveric, and dermatomal studies. In anatomical studies, it can be said that individual variability in anatomical structures may be a factor, and demographic characteristics may also affect this situation. In a cadaver study by Aponte et al. [33], the fact that dye spread in different dermatomes in both hemithorax even in the bilaterally applied block suggests that there may be changes even in the same individual. In addition, case-based cadaveric studies have reported anatomical variations in chest wall muscles [34–36]. It was emphasized that these variations may cause complications especially in surgical applications and clinicians should be cautious in this regard. It can be thought that this situation may also be effective in the spread of LA. Considering the results of all these limited studies, we think that the Bilevel-ESPB application may provide more effective analgesia. Moreover, the possible insufficient spread can be limited by a second injection at a different level. Although dermatomal spread was not evaluated in our study, the more effective analgesia level provided in the Bi-Level group in the early postoperative period may explain this situation. Large-scale cadaveric, dermatomal analysis and dye studies to be carried out on this subject will be important in terms of clarifying the issue.

### Limitations

There are some limitations in this study. First of all, this was a pilot trial with a small sample size, and the results must be confirmed in greater trials. Secondly, considering the patient’s comfort, dermatomal evaluation could not be performed because the blocks were performed under general anesthesia before the surgical incision. However, sedo-analgesia applied to prevent pain and anxiety in patients may limit this evaluation, even if it is performed in an awake patient. Fourthly, Since the feeling of pain is a subjective condition, the results may vary from person to person. In addition, the use of the PCA device varies individually. Moreover, the limited effect of opioids on pain, especially while coughing, may also affect the pain condition. Finally, in this two-center study, although the block was applied by anesthetists with US experience, this situation has the potential to affect the effectiveness of the operator-based block.

## Conclusions

As a result, ESPB has many advantages in terms of ease of application and effective analgesia, and studies conducted in recent years support this situation. However, it still carries many question marks such as the volume and level to be applied. Although there are limited studies, it is stated that Bi-Level ESPB made at different levels provides effective analgesia. In our study, the fact that more effective analgesia was achieved in the early postoperative period in the group treated with Bi-Level ESPB supports this situation. However, there is a need for large-scale studies on this subject.

## Data Availability

The datasets generated during and/or analyzed during the current study are available from the corresponding author on reasonable request.

## Abbreviations

ASA: American Society of Anesthesiologists
BMI: Body mass index
ERAS: Enhanced recovery after surgery
ESPB: Erector spinae plane block
IV: Intravenously
LA: Local anesthetic
LAST: Local anesthetic systemic toxicity
PCA: Patient-controlled analgesia
T: Thoracal
TPVB: Thoracic paravertebral block
US: Ultrasound
VAS: Visual analog scale
VASr: Visual analog scale scores at rest
VASc: Visual analog scale scores at cough
VATS: Video-assisted thoracic surgery
